# Health Advice from Internet Discussion Forums: How Bad Is Dangerous?

**DOI:** 10.2196/jmir.5051

**Published:** 2016-01-06

**Authors:** Jennifer Cole, Chris Watkins, Dorothea Kleine

**Affiliations:** ^1^ H2B2 Department of Computer Science Royal Holloway, University of London Egham, Surrey United Kingdom; ^2^ Department of Computer Science Royal Holloway University of London London United Kingdom; ^3^ ICT4D Centre Department of Geography Royal Holloway, University of London London United Kingdom

**Keywords:** eHealth, communication, Internet, social media, health literacy, health care information systems, information seeking, information science, Medicine 2.0, Web-based and mobile health interventions

## Abstract

**Background:**

Concerns over online health information–seeking behavior point to the potential harm incorrect, incomplete, or biased information may cause. However, systematic reviews of health information have found few examples of documented harm that can be directly attributed to poor quality information found online.

**Objective:**

The aim of this study was to improve our understanding of the quality and quality characteristics of information found in online discussion forum websites so that their likely value as a peer-to-peer health information–sharing platform could be assessed.

**Methods:**

A total of 25 health discussion threads were selected across 3 websites (Reddit, Mumsnet, and Patient) covering 3 health conditions (human immunodeficiency virus [HIV], diabetes, and chickenpox). Assessors were asked to rate information found in the discussion threads according to 5 criteria: accuracy, completeness, how sensible the replies were, how they thought the questioner would act, and how useful they thought the questioner would find the replies.

**Results:**

In all, 78 fully completed assessments were returned by 17 individuals (8 were qualified medical doctors, 9 were not). When the ratings awarded in the assessments were analyzed, 25 of the assessments placed the discussion threads in the highest possible score band rating them between 5 and 10 overall, 38 rated them between 11 and 15, 12 rated them between 16 and 20, and 3 placed the discussion thread they assessed in the lowest rating band (21-25). This suggests that health threads on Internet discussion forum websites are more likely than not (by a factor of 4:1) to contain information of high or reasonably high quality. Extremely poor information is rare; the lowest available assessment rating was awarded only 11 times out of a possible 353, whereas the highest was awarded 54 times. Only 3 of 78 fully completed assessments rated a discussion thread in the lowest possible overall band of 21 to 25, whereas 25 of 78 rated it in the highest of 5 to 10. Quality assessments differed depending on the health condition (chickenpox appeared 17 times in the 20 lowest-rated threads, HIV twice, and diabetes once). Although assessors tended to agree on which discussion threads contained good quality information, what constituted poor quality information appeared to be more subjective.

**Conclusions:**

Most of the information assessed in this study was considered by qualified medical doctors and nonmedically qualified respondents to be of reasonably good quality. Although a small amount of information was assessed as poor, not all respondents agreed that the original questioner would have been led to act inappropriately based on the information presented. This suggests that discussion forum websites may be a useful platform through which people can ask health-related questions and receive answers of acceptable quality.

## Introduction

### Background

Over the past 2 decades in England and Wales, consultation rates within general practitioners’ (GP) surgeries have increased from approximately 220 million in 1995 to 300 million in 2008 and are estimated currently at approximately 340 million [1]. Over the last decade, the number of attendances at accident and emergency (A&E) units in the National Health Service has increased more than 30%, from 14 million per year prior to 2003/2004 to 21.7 million in 2013/2014, and numbers are continuing to grow [2]. Pressure on GP surgeries may be one of the reasons for the increasing pressure on hospital A&E departments: 22% of patients report that it is not easy to get through to their GP’s surgery on the telephone and 9.8% of people who are unable to get a convenient GP appointment go to an A&E walk-in center instead [1].

Since 2008, online health-seeking information in the United Kingdom has increased dramatically, from 18% of UK adults saying they looked for health information online in 2008 to 43% in 2013, with an increase of 59% among the 25 to 29 years age group [3]. Health information seeking represented one of the fastest growing areas of Internet use measured by the UK government during the period from 2008 to 2013 and in 2014; 8% of people aged 16 to 35 years and 15% of those aged 55 to 64 years made a GP appointment using the Internet. The United Kingdom is the second highest country globally for Internet health searches; in a recent survey, “Google my symptoms” was a more common first action than “book a doctor’s appointment” or “visit a pharmacy for advice” [4]. In 2014, the number of health searches carried out in the United Kingdom increased by 19% [4].

### Quality Considerations for Online Health-Seeking Behavior

Supporting individuals to shift at least some of their health-seeking behavior from a face-to-face consultation with a medically qualified practitioner to seeking information online, both before and following diagnosis, provides opportunities to relieve the pressure on GP surgeries and A&E departments. However, it is also dependent on the information found online being of sufficiently high quality that following it does not pose a health risk. Prior studies of health information online have shown that it is of variable quality [5-10]. Although much concern has been expressed over this [11-15], few examples of actual rather than potential harm have been documented [16,17].

Internet users often seek disease-specific information [18,19], including information that will enable them to diagnose a particular health problem [20]. Because trusted brands play an important role in health-seeking behavior [21-23], one way to make health-seeking behavior more comfortable for the Internet user may be to encourage them to turn to known and trusted websites when seeking health information, leveraging trusted brands to help them feel confident about the information they find there. If the brand is not health-specific, but is a source of information on a range of topics that the patient already trusts, they may be more likely to turn to it for information when they engage in online health-seeking behavior for the first time. Respondents to a short study on health information-seeking behavior during the 2014-2015 Ebola crisis in West Africa largely did not begin to use new modes of communication to seek out health information. Instead, they searched for health information through platforms and media they were already familiar with, turning first to trusted health and information brands, such as the World Health Organization, the BBC, and government ministries of health in addition to knowledgeable friends [24].

### Characteristics to Support Health-Seeking Behavior

Online discussion forums have a number of characteristics that could benefit online health information seekers. Discussion is known to enable better learning and absorption of knowledge [25,26] and this has been identified as a benefit of discussion forums in general [27] and of online discussion forums specifically [28]. The emergence of Web 2.0 has provided new opportunities to gain and share knowledge about health issues [29-31] and discussion forums display positive attributes relating to all 4 website characteristics (source, medium, message, and receiver) that have been identified as important to engendering trust during online health-seeking behavior [32]. In particular, discussion forums can act as both the medium for and source of health information. Because both doctors and friends can be accessed through online discussion forums, the Internet should not be seen as a competing category to face-to-face interaction with such sources, but rather an enabler of it.

A weakness of the existing literature is the tendency to approach the Internet as if it is a homogenous environment where every website can be trusted or mistrusted equally until trust is added on by accreditation seals or source authority. This does not consider whether some characteristics, such as voted discussion forums that offer the ability to counter a previous post with more accurate information or to fill-in the information missing from a previous incomplete answer, make discussion forums inherently more conducive to the transfer of good quality information than other types of websites. All spaces exert influences on the choices that people make in those spaces. The more designers, owners, operators, and users of online discussion forums are aware of what these influences are likely to be, the more able they will be to consider how they can influence users’ choices [33,34].

The aim of this study is to provide an assessment of the quality and quality characteristics of information found in online discussion forums so that doctors, patients, and health care policymakers can better understand the online discussion forum environment and the information found there.

## Methods

### Selection Criteria

Our study involved UK-qualified medical doctors and UK (London)-based nonmedically qualified individuals assessing the information found in 3 online discussion forums (Reddit [35], Mumsnet [36], and Patient [37]) relating to 3 health conditions. We selected 3 health conditions that affect a high number of individuals in the United Kingdom: diabetes, chickenpox, and human immunodeficiency virus (HIV). According to the most recent figures from Public Health England, an estimated 107,800 individuals in the United Kingdom were living with HIV in 2013 [38]. An estimated 3.2 million (7%) of the UK population is living with diabetes [39], of whom 10% have type 1 diabetes and the remaining 90% have type 2. An estimated 90% of all Britons will have had chickenpox by the age of 15 [40], although no exact figures on infection exist for the United Kingdom because not all cases receive clinical attention. It is important to note that although US health policy positions chickenpox as a dangerous disease for which childhood vaccination is recommended [41], this is not the case in the United Kingdom, where it is positioned as a mild childhood disease for which vaccination is only necessary for high-risk groups. Professional medical consultation is considered necessary only in cases of complications listed on the website of the UK National Health Service [42].

There is evidence that a high volume of health-seeking information occurs in relation to all 3 conditions. Diabetes and HIV both feature in the top 10 most searched for diseases on Google (diabetes at number 2 with more than 9 million monthly global searches in 2013, HIV at number 4 with more than 6 million, and acquired immune deficiency syndrome [AIDS] at number 6 with 5 million) [43]. Although chickenpox appears lower on the list (at 43 with more than half a million global monthly searches), it is one of only a handful of communicable diseases found there and is the most significant childhood disease in the United Kingdom. Vaccination is uncommon (ie, the majority of the population are likely to catch it), but many parents do seek professional advice when their children develop symptoms. Therefore, it was likely that a considerable volume of health information would exist online for these 3 conditions and that forums where health is discussed were likely to have discussion threads related to them.

### Selection of Discussion Forum Websites and Discussion Forum Threads

Three online discussion forum websites were selected based on their popularity and common usage by the UK population (rather than among specialist interest groups or social media superusers). These included 2 general discussion websites (Reddit and Mumsnet) and a health-specific site (Patient). We investigated each of the 3 websites to see if their message forums had existing discussion threads related to these conditions and found that all 3 health conditions were discussed on all 3 forums.

We selected specific discussion threads for the survey subjectively by undertaking a basic search inside each selected website on the chosen health conditions between February 15 and 17, 2015, and reading through the returned results to find questions for which we felt the original poster could (and probably should) have sought advice from a qualified medical practitioner. Discussion threads were rejected if the question did not require a medical or scientific reply (eg, a diabetic asking others whether they thought disclosing his diabetes on job applications would be disadvantageous) and if the question had received less than 2 replies. We selected 25 suitable questions (Reddit: n=9; Mumsnet: n=8; Patient: n=8; diabetes: n=8; HIV: n=9; chickenpox: n=8) according to the order they appeared in the search results, favoring discussions in which the question had been posted within the previous 12 months. If no suitable questions matching these criteria appeared within the first 50 search results, questions posted earlier or from beyond the first 50 search results were selected. Eleven of 25 questions selected appeared to be prediagnosis and were asking if symptoms they or a friend/family member were exhibiting might be indicative of a certain condition (eg, diabetes), 9 of 25 appeared to be postdiagnosis asking for advice on how to act in light of a condition (eg, whether certain exercise routines were suitable for diabetics), and 4 were asking for general advice on topics such as vaccination. Each question and the discussion thread that followed it was then assessed by more than one assessor. In total, 79 assessments were returned (mean 3.2, range 2-7 for the 25 questions).

### Selection of Study Participants

We aimed to have the information in the forums assessed for quality by UK-qualified medical doctors (1 GP, 2 hospital infection specialists, 1 hospital-based diabetes consultant, 4 who did not give exact details) and also by London-based individuals who were not medically qualified, but who had experience with the health issue being discussed as a patient or as a carer of a patient.

The majority of the doctors were recruited through Ashford and St Peter’s Hospital, which has links with the Health, Human Body and Behaviour (H2B2) program at Royal Holloway, University of London. Two other medical doctors, known personally by the authors, were also invited to participate.

The nonmedically qualified participants for diabetes were recruited by contacting the chairs of 2 (offline) support groups for diabetics. Contact addresses for support groups are given on the website of the diabetes support charity Diabetes UK, which enabled group coordinators to be contacted personally and asked to take part. For chickenpox, parents of children in the common age group for contracting chickenpox (age 2-10 years) were recruited through the Parents and Friends Associations of 2 local West London schools (Lovelace Primary School in Chessington and Putney Girls High School). The Terrence Higgins Trust, a charity that supports people living with AIDS and HIV, was also approached and asked to contact people living with HIV who would be willing to take part, but they did not reply. As such, no HIV-positive patients participated and the questions relating to HIV were answered by doctors only. Participants were self-selecting and, therefore, may be subject to selection bias. Demographic data collected on the participants was minimal: it recorded whether or not they were medically qualified and confirmed they were adults older than age 18 years before taking part, but no other particulars were recorded because these were not deemed necessary for this part of the study. Participants were given the option of taking part anonymously; 12 chose to disclose no other information than their level of qualification. Five of the doctors, but only one of the nonmedically qualified participants, provided a contact email address.

Participants were sent, by email, a list of paired URL links for each discussion thread they were asked to assess. One linked to the actual online forum discussion thread, which they saw in situ with no modification made to it for the sake of the study, and the other to an online assessment form. Each discussion thread was assessed against the same criteria. The discussion threads were assessed according to 5 criteria and the participant responded by rating the information from highest quality to lowest quality (range 1-5) on:

1. The medical/scientific accuracy of the information found there [5,7,9];
2. The medical/scientific completeness of the information [9,10,44];
3. How sensible they considered the answers provided to be;
4. Whether they thought someone reading the website would act appropriately based on the information provided; and
5. How useful they felt the answers given would be to the original poster.

An additional question was asked to check that the respondents found the discussions easy to follow; only 4 assessments recorded any level of difficulty in following the discussions.

The responses assessed perceived factual quality of the answer (accuracy and completeness), gave a subjective assessment on that information (how sensible was it?), and subjective assessments of how the reader might respond (would they act appropriately and would they find the information useful?). We included this differentiation in the questions because, although many previous studies have criticized online health information for being of poor or variable quality [11,14,15], far less have found actual evidence of poor information leading to inappropriate or dangerous health decisions being made [16,17,45,46]. Because even fewer studies focused on how likely it is that Internet discussion forum readers will take action based on the information they found there, exploring perceptions around this is of particular interest.

In each assessment, the discussion threads could be assigned 1 of 5 rating values, for which the highest (1) related to the best quality information and the lowest (5) to information that was considered to be inaccurate or ill advised. The criteria for marking were consistent across each health topic and website, and provided a potential overall score of between 5 (5*1, top rating for each criteria) and 25 (5*5, lowest rating for each criteria) to each discussion forum thread.

Participants were invited to participate between May 12 and June 4, 2015, and were given 2 to 3 weeks to reply. The final survey assessments were accepted on June 13, 2015. Participants were sent a mean 8 (range 7 to 25) discussion threads to assess (each of which required assessments of the 5 separate criteria) based on their particular area of experience or medical expertise, with only one participant—a recently retired GP—offered all surveys to complete. The assessments were completed, and results collected, using the free online survey software SmartSurvey. A generic version of the assessment questionnaire is available in Multimedia Appendix 1.

## Results

### Survey Data Returned

A total of 79 assessments of discussion threads were returned at least partially completed (as of June 13, 2015). For 78 assessments, all 5 criteria were assessed and rated, but on 1 assessment, 2 of the criteria were skipped. Seventeen separate individuals took part*,* 8 of whom identified themselves as medically qualified. The qualified medical doctors completed 58 of 79 (73%) returned surveys and 21 of 79 (27%) surveys were completed by the nonmedically qualified respondents.

**Figure 1:**
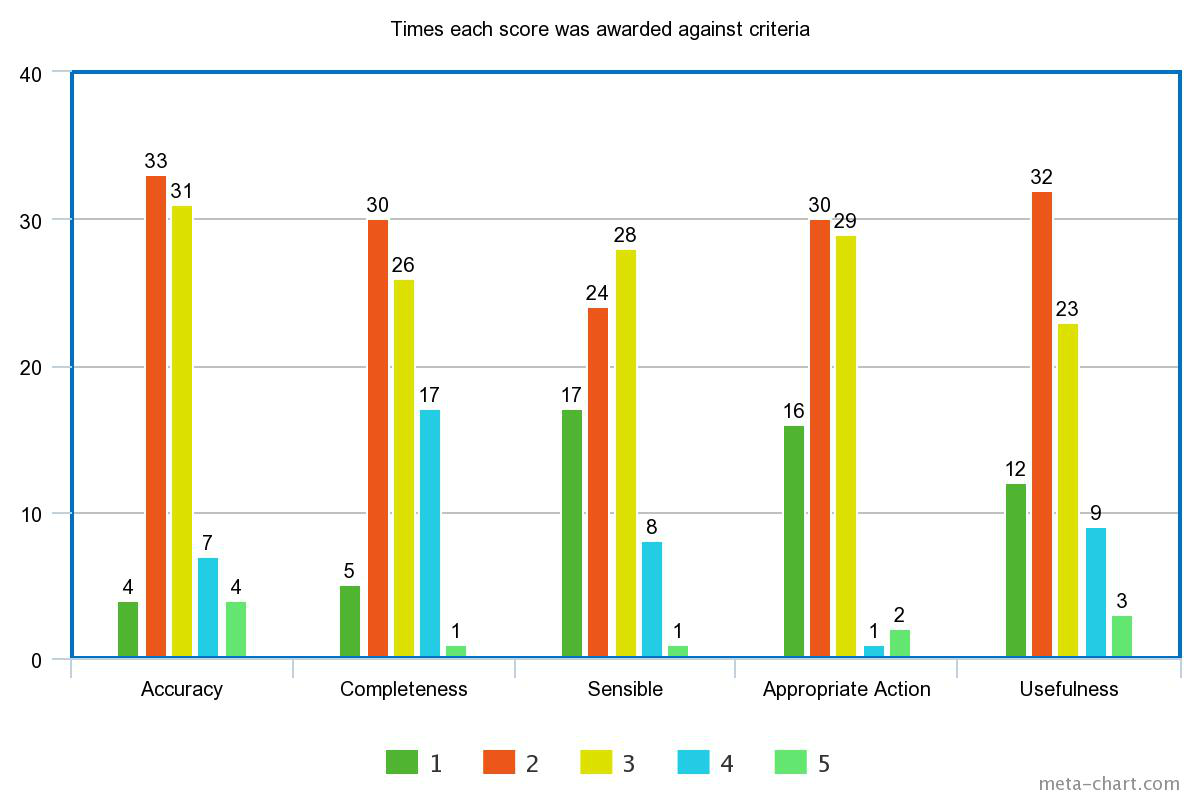
Comparison of ratings (scores) awarded to each category across all websites and health conditions.

Tables 1-3 show the data from all survey responses by health condition and website. Multimedia Appendix 2 includes a visual representation of the quality scores and a reference to the actual question as it appeared on the Internet discussion forum website (as the assessors saw it when they made their assessment). Figure 1 shows a visual comparison of ratings in each category across all websites and health conditions.

**Table 1:** Quality score data from all survey responses for diabetes-related questions.

Website, question, and respondent			Quality score (1=high, 5=low)					Total
			Accurate	Complete	Sensible	Appropriate	Useful	
**Reddit**								
	**Q1. First party since being diagnosed, need advice?**							
		Doctor 1	2	1	1	1	1	6
		Doctor 2	2	2	1	1	1	7
		Public 9	2	2	1	2	2	9
	**Q2. Advice for exercise and midnight lows?**							
		Doctor 1	2	3	3	2	2	12
		Doctor 2	2	2	1	1	1	7
**Mumsnet**								
	**Q3. Are anger outbursts normal with diabetes?**							
		Doctor 1	2	3	2	2	2	11
		Doctor 2	3	3	2	2	2	12
		Public 1	2	2	2	2	2	10
		Public 9	1	4	3	3	2	13
	**Q4. Signs of diabetes or paranoid Mummy?**							
		Doctor 1	2	2	2	3	3	12
		Doctor 2	2	2	1	1	1	7
		Public 1	2	3	2	2	2	11
**Patient**								
	**Q5. Longer to get over a cold with diabetes?**							
		Doctor 1	5	4	4	3	3	19
		Doctor 3	3	3	2	2	2	12
		Public 2	3	1	3	3	4	14
		Public 3	4	3	3	2	2	14
	**Q6. Can this be diabetes?**							
		Doctor 1	2	3	3	3	3	14
		Public 2	3	1	3	2	4	13
	**Q7. Do I have Type 1 diabetes?**							
		Doctor 1	2	2	1	1	1	7
		Doctor 2	2	2	1	1	1	7
		Public 1	2	2	2	2	2	10
	**Q8. Diabetes: advice please?**							
		Doctor 1	2	3	2	3	2	12
		Public 2	3	1	—^a^	—^a^	4	—

^a^ Scores are missing because respondent did not answer for these criteria.

**Table 2:** Quality score data from all survey responses for HIV-related questions.

Website, question, and respondent			Quality score (1=high, 5=low)					Total
			Accurate	Complete	Sensible	Appropriate	Useful	
**Reddit**								
	**Q9. I found out I had HIV; not clear about the stage**							
		Doctor 1	2	3	2	2	2	11
		Doctor 4	2	2	2	2	2	10
	**Q10. HIV and depression**							
		Doctor 1	3	5	4	4	5	21
		Doctor 4	3	4	3	3	2	15
	**Q11. Question about HIV and personal fitness**							
		Doctor 1	2	2	2	1	1	8
		Doctor 4	2	1	1	1	1	6
	**Q12. FAQ: Worried? Risk, testing, and anxiety**							
		Doctor 1	1	2	1	1	2	7
		Doctor 4	1	2	1	2	1	7
**Mumsnet**								
	**Q13. Babysitter has just announced he’s HIV positive**							
		Doctor 1	3	2	3	3	4	15
		Doctor 4	3	3	2	2	2	12
	**Q14. Question about HIV (and partner)**							
		Doctor 1	3	2	3	3	3	14
		Doctor 4	2	3	2	2	2	11
	**Q15. Children and HIV**							
		Doctor 1	3	2	3	2	3	13
		Doctor 4	2	4	2	3	3	14
**Patient**								
	**Q16. HIV question**							
		Doctor 1	2	2	2	2	2	10
		Doctor 4	2	3	1	2	2	10
	**Q17. HIV infection: intestinal yeast after 4 months?**							
		Doctor 1	3	3	3	3	4	16
		Doctor 4	4	3	3	2	2	14

**Table 3:** Quality score data from all survey responses for chicken pox–related questions.

Website, question, and respondent			Quality score (1=high, 5=low)					Total
			Accurate	Complete	Sensible	Appropriate	Useful	
**Reddit**								
	**Q18. Is this chickenpox? Help!!**							
		Doctor 1	5	4	4	5	5	23
		Doctor 4	3	3	3	2	2	13
		Doctor 5	4	4	3	3	4	18
		Doctor 6	5	4	4	2	3	18
		Public 4	3	4	3	2	3	15
		Public 5	4	2	2	2	2	12
		Public 6	3	2	2	1	2	10
	**Q19. Did you give your child the chickenpox vaccine?**							
		Doctor 1	2	2	2	1	2	9
		Doctor 4	2	2	1	2	1	8
		Doctor 5	2	2	1	1	1	7
		Doctor 6	3	3	4	2	3	15
		Public 4	3	3	3	3	3	15
		Public 5	2	2	1	1	1	7
		Public 6	3	4	3	3	3	16
	**Q20. Chickenpox: why more dangerous to adults?**							
		Doctor 1	3	3	3	3	4	16
		Doctor 5	3	4	3	3	4	17
		Doctor 7	3	3	3	3	3	15
	**Q21. Chickenpox: is 5 months too young to expose?**							
		Doctor 1	3	3	3	3	3	15
		Doctor 4	2	2	2	2	2	10
		Doctor 5	5	4	4	5	5	23
		Public 4	3	3	3	3	3	15
		Public 7	3	3	2	2	2	12
		Public 8	4	4	4	3	3	18
	**Q22. Has your child had the chickenpox vaccine?**							
		Doctor 1	3	4	3	3	3	16
		Doctor 4	2	2	2	1	2	9
		Doctor 5	3	3	3	3	3	15
		Public 6	3	4	3	3	3	16
		Public 7	3	2	4	3	3	15
	**Q23. Is it normal to be so very ill with chickenpox?**							
		Doctor 1	4	4	3	2	3	16
		Doctor 4	2	2	3	1	2	10
		Doctor 8	2	2	1	1	2	8
		Public 4	3	3	1	3	3	13
	**Q24. Should toddler get the chickenpox vaccine?**							
		Doctor 1	2	2	2	3	3	12
		Doctor 4	2	3	2	2	2	11
		Doctor 5	1	2	1	3	4	11
	**Q25. Strange symptom with chickenpox**							
		Doctor 1	4	4	4	2	4	18
		Doctor 4	3	3	3	2	2	13
		Doctor 5	3	4	2	3	2	14

On average (excluding the retired GP), medically qualified respondents completed 5 surveys each and nonmedically qualified respondents completed 2 to 3 assessments each. When asked, the reason why respondents did not complete all assessments they were offered was lack of time. In total, this provided 393 criteria ratings across all 79 assessments (see Table 4).

**Table 4:** Number of individual criteria assessed (5 assessments per discussion forum thread) out of 393.

Condition	Discussion forum, n			Total, n
	Reddit	Mumsnet	Patient	
Diabetes	25	35	53^a^	113
HIV	40	30	20	90
Chickenpox	85	75	30	190
Total	150	140	103	393

^a^ Two questions on a survey were skipped.

Of the 353 assessments made overall, assessors rated the majority as a score of 2 (n=149) or 3 (n=137) on the scale of 1 to 5. Some information was rated the highest score of 1 (n=54) and a smaller proportion was rated the lowest scores (n=42 for a score of 4; n=11 for a score of 5). The lowest possible rating was given only 11 times out of 393 (2.8%) across the entire survey in comparison to 54 instances (13.5%) in which the highest possible rating was given. No discussion thread was given the lowest possible rating across all 5 categories (which would have given it an overall score of 25 suggesting that the information was inaccurate, incomplete, and likely to lead to harm) or the highest possible rating (which would have given it an overall score of 5 suggesting that the information was entirely accurate, complete, and would lead to appropriate action being taken).

When ratings were considered across all 5 criteria on which discussion forums were assessed and grouped into 4 value groups (5-10: threads were predominantly given 1 of 2 highest ratings for all criteria; 11-15: threads were largely given the high-middle ratings; 16-20: threads were often rated in the lower categories; 20-25: threads were assessed poorly on all 4 criteria) across the 79 surveys completed, 25 scored between 5 and 10, 38 scored between 11 and 15, 12 scored between 16 and 20, and 3 scored between 21 and 25. The one assessment that was only partially completed and could not be awarded an overall score was excluded from this stage.

High ratings were awarded more often than low ratings by a factor of 4:1. Ratings of 1 or 2 were awarded 203 of 393 times (51.7%), whereas ratings of 4 or 5 were awarded 53 of 393 times (13.5%). Overall ratings of 5 to 10 or 11 to 15 were awarded 63 of 78 times (81%), whereas ratings of 16 to 20 or 21 to 25 were awarded 15 of 78 times (19%).

### How Is “Bad” Information “Bad”?

Previous studies tended to assess information against a single criterion for quality (ie, completeness of information or accuracy of information) across all websites available regardless of their characteristics. This study enabled a comparison across a number of criteria and considered how website characteristics specific to discussion forums may influence this.

Although 18 of 79 surveys (23%) were considered to be incomplete (receiving scores of 4 or 5), covering “very little” or “none” of the information the assessor would expect to see, a smaller number than this (11/79, 15% ) considered the information given to be “somewhat” or “very” medically inaccurate (receiving scores of 4 or 5 for those criteria). The findings are consistent with the existing literature in that information marked on quality is generally more related to the incompleteness of the information rather than actual inaccuracy [5,10,45]. An even smaller number (3/79, 4%) thought that poor information may lead to someone acting in a way that may put their health at risk. The results suggest that even if information is considered to be inaccurate or incomplete, this may not necessarily result in poor advice being given.

Because all message threads were rated by at least 2 assessors, and some were rated by 5 to 7 different assessors, this offered an opportunity to note whether information assessed as poor was marked consistently (or inconsistently) by all assessors, which would suggest an element of subjectivity in the assessment. In general, assessors scored consistently. For example, Q1 was awarded overall scores of 6, 7, and 9 by its 3 assessors; Q3 scored 10, 11, 12, and 13; and Q20 scored 15, 16, and 17. Some threads scored consistently high by all assessors (eg, Q1, Q7, Q12) and some scored consistently average (Q3, Q20, Q25). No discussion thread was consistently awarded the lowest band range by all its assessors.

Assessors who marked more than 3 discussion threads awarded marks reasonably evenly across the possible rating bands: Doctor 1, the only respondent who assessed all 25 threads, gave ratings ranging from 6 to 24; Doctor 4 gave a range from 6 to 15; and Doctor 6 gave a range from 7 to 23. The nonmedically qualified respondents tended to be more cautious awarding ratings marks; they awarded across a range of 7 to 18. Only 1 of the 10 lowest-rating assessments was returned by a nonmedically qualified respondent (Q21/Public 8). This could be seen as nonmedically qualified respondents being less able to recognize poor quality information or less confident about highlighting it as poor quality. However, the nonmedically qualified respondents tended to agree with the medically qualified respondents when assessing information as being inaccurate and/or incomplete, but they gave different responses on how likely someone would be to act inappropriately on the information provided (eg, see Q21/Doctor 5 and Public 8).

In the 11 cases in which the lowest possible rating was returned for a discussion thread, other assessors (including doctors) of the same thread rated it more favorably. If a thread was rated in the highest score group (5-10) by one respondent, the lowest rating it received from any of the other assessors was 16, with the largest range 7 to 16 (Q19), whereas the 3 threads that rated in the lowest band (21-25) received a much broader range of scores (Q18: 10-24; Q21: 10-23). When information was considered to be of middling quality overall, it was more likely that some assessors would consider it to be very poor. However, in most cases, low ratings were outliers in a broad range; for instance, the low rating of 23 awarded to Q21 by assessor Doctor 5 and the rating of 24 awarded to Q18 by assessor Doctor 1 were outliers to a range of 10 to 18 in both cases from the same thread’s other assessors.

### Assessment of Quality by Message Forum

There was some variation in quality between the message forums, with Reddit containing the highest quality information (rating of 1: n=33) more often than either Mumsnet (n=9) or Patient (n=12), but also being more likely to contain the lowest quality information (rating of 5: Reddit=7; Mumsnet=3; Patient=1) than the other 2 websites.

### Assessment of Quality by Health Condition

Message threads related to chickenpox were less likely to be awarded high ratings (score of 1 or 2) than discussion threads related to either HIV or diabetes. The middle rating (score of 3) was most often awarded to discussions on chickenpox, whereas both HIV and diabetes were mostly likely to be rated 2. Eight of the total 11 lowest ratings were awarded against chickenpox discussion threads and 8 of the 10 lowest-rated threads overall were chickenpox threads.

**Figure 2:**
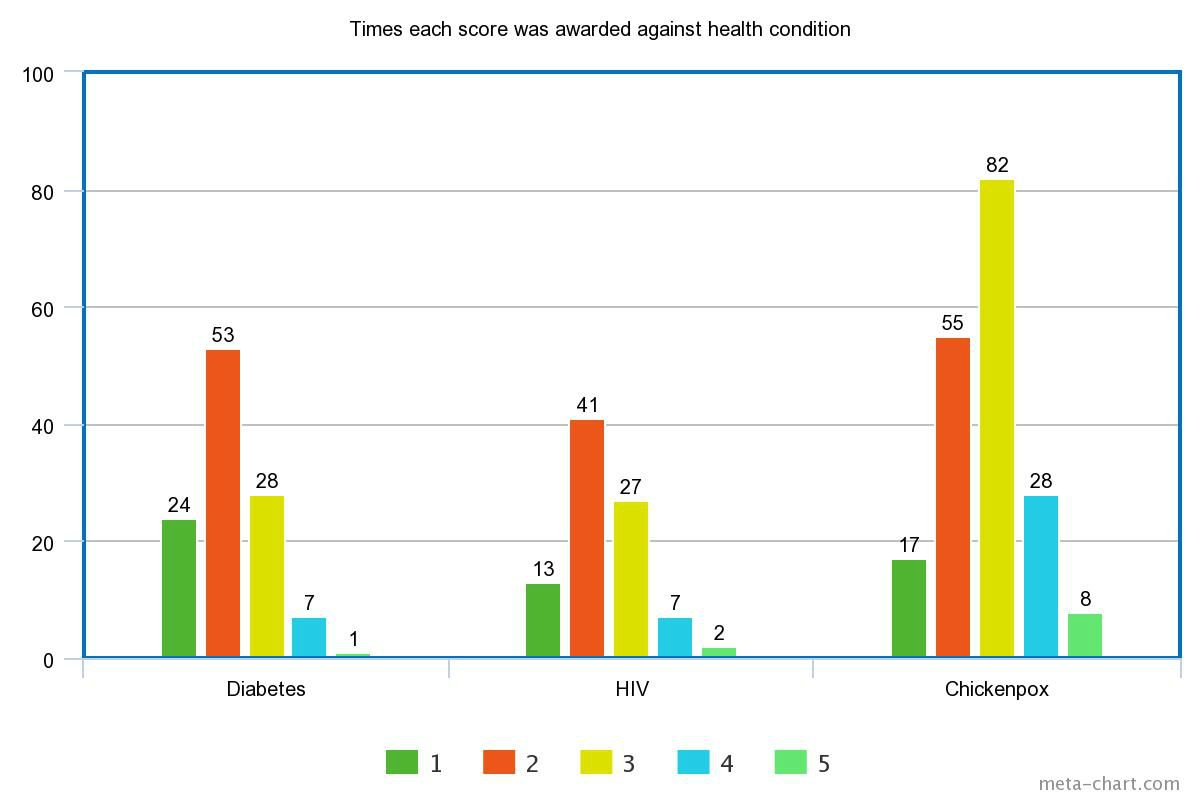
Overall ratings (scores) awarded by health condition.

Figure 2 displays all scores of the individual health topics to show the variation in results across the different health conditions.

## Discussion

### Principal Results

The results of this survey suggest that, in general, the health information found in discussion forums is of reasonably good quality and only rarely does it contain information that is very inaccurate (4/79) and which some reviewers (3/79) feel may lead someone to act in a way that may put their health at risk.

These results are broadly consistent with those found elsewhere in the existing literature on the completeness and accuracy of health information found online. Previous studies on a diverse range of health conditions, including cancer, managing fever in children, and childbirth, have consistently suggested that approximately 60% to 70% of information is generally of good quality [5,10,15] with only approximately 5% to 7% considered genuinely inaccurate [5,15,45]. The results also suggest that people may be more able to make sensible decisions when faced with poor quality information than doctors give them credit for. This warrants further study.

Rating the discussion threads on different criteria enables us to look more closely than previous studies at how, where, and why poor ratings are awarded. It is interesting to note, for example, that the controversial discussions around vaccination and herbal/natural remedies in the chickenpox discussions led to 36 separate low ratings of 4 or 5 being awarded. These were more often awarded against the inaccuracy of the information (n=11) or incompleteness (n=18) than the information being likely to lead the poster to make a somewhat inappropriate or very ill-advised decision (n=3).

It was not within the scope of the study to compare the questions that appeared to be asked prediagnosis with those that appeared to have been asked postdiagnosis. It was also not within the scope to compare those questions that were asked about the more serious conditions (ie, HIV and diabetes) with those that were asked about the milder condition (ie, chickenpox). We appreciate that these may be important factors in influencing the replies given and they warrant further research.

### Q18. Reddit/Chickenpox

The discussion thread that was rated most poorly was Q18 (“Is this Chickenpox? Help!!”) for chickenpox on Reddit, on which a parent had posted a photo of spots their child had developed and asked, “Is this chickenpox?” Two respondents considered the information given to be very medically/scientifically inaccurate, one of whom also considered the information to be very ill advised, likely to lead the questioner to make a very ill-advised decision, and to act in a way that may put their health in danger.

In total, 8 respondents completed this questionnaire. Although more than half (4/7) considered the information given to be “somewhat” or “very” scientifically inaccurate and to cover “very little” of the medical information they would expect to see, 6 of 7 respondents did not think this would actually lead to harmful behavior. It is also worth noting that some posters did encourage the original questioner to go to the doctor, who later posted an update to say that they had taken this course of action. This is particularly interesting because it provides proof that although the information was assessed by some experts to be poor, it did not lead to dangerous behavior and the original poster was capable of sorting the sensible advice from the mix of replies given.

### Q21. Mumsnet/Chickenpox

On Mumsnet, Q21 (“Chicken pox–is 5 months too young to expose?”) for chickenpox returned 3 lowest possible ratings against the inaccuracy of the information and in the 2 categories relating to how the poster might act. The survey was completed by 6 respondents in total and the low scores were awarded by only 1 of the 6; the other 5 rated the information more favorably. The discussion related to a parent’s question about the safety of exposing their 5-month-old child to someone who was infected with chickenpox in the hope of getting the disease “out of the way.” The discussion contained a range of views, from some parents who thought there would be little harm in it (largely due to experience of their own children having had the disease at a similar age with no problems arising) to those who considered it dangerous. Several replies actively discouraged the parent from exposing an infant so young. None of the discussions displayed antivaccination viewpoints and none actively encouraged the mother to go ahead. At the end of the discussions, the original poster summarized her understanding of the discussions and stated that, after reading the advice, she thought that the ideal age to catch chickenpox “is 2 to 6 years,” suggesting that she had been convinced that deliberately exposing a 5-month-old child would not be a good idea. Therefore, it is difficult to understand why one assessor felt that the poster would have made an ill-advised decision that would have put [her child’s] health at risk, rather than taking the view of another assessor who made the qualitative comment: “I think she came to the right conclusion based on the information given.”

### Q10. Reddit/HIV

Two lowest ratings were given in response to Q10 (“I am somewhat prone to depression, but even more so now that I am HIV+. How do you guys deal with it?”) for HIV on Reddit, which asked about links between HIV diagnosis and depression. One respondent ranked this discussion in the lowest categories for completeness of information and how the poster might act based on information provided. A qualitative response given in the comments box explained that the low ratings had been given because none of the replies encouraged the poster to seek professional help, which the respondent (a GP) believed they needed. Therefore, it was not so much that poor information was given, but that the appropriate good information was not. Another respondent (a hospital consultant) marked the discussion more favorably.

### Q5. Patient/Diabetes

The final low rating was recorded against a discussion on whether diabetes affects a person’s ability to recover from a cold (“Does it take longer to get over a cold if you have type 2 diabetes?”). One of 4 assessors felt that the information given was “very medically/scientifically inaccurate,” but in this case, they did not feel that the information would lead the poster to make an ill-advised decision or to act in a way that may put their health at risk.

Because 4 of 79 surveys were responsible for all 11 instances of low ratings and just one of those (Q18. Reddit/chickenpox) was responsible for 5 of 11 low ratings (see Table 5), this warrants further research.

**Table 5:** Discussion forums returning the lowest possible ratings.

Header	Inaccurate	Incomplete	Ill advised	Make bad decision	Dangerous to health
Q5. Patient/diabetes	1				
Q10. Reddit/HIV		1			1
Q18. Reddit/chickenpox	2		1	1	1
Q21. Mumsnet/chickenpox	1			1	1

There was no discussion thread that was consistently rated in the lowest (or even lowest plus second lowest) categories by all its respondents, suggesting that what constitutes poor information is as much a subjective judgment on the part of the reviewer as an absolute. Respondents disagreed more on how people who read the information may act based on it than on the accuracy or completeness of the information. Previous studies have suggested that there is an element of subjectivity attached to assessments of quality [45] and although the results of this study uphold this, further exploration is warranted of how health information in online discussion forums is received and acted on.

### Limitations of the Study

A number of limitations have to be taken into account when considering the results presented here. Firstly, the sample size was very small, consisting of only 17 individuals from a limited demographic (UK adults in West London). This cannot be considered to be representative; a much larger sample would need to be surveyed to ensure results could be applied more generally.

Secondly, recruiting respondents to the study was difficult, especially recruiting nonmedically qualified respondents. The diabetes support groups contacted were nervous about involving their members in a study that may direct them to incorrect and potentially harmful information. The HIV support charities contacted did not respond. Although it was easier to recruit parents to assess the chickenpox discussion, the numbers recruited were still fewer than hoped for. A larger future study would need to consider more efficient ways of recruiting higher numbers of participants.

### Comparison With Prior Work

These results support other studies of online health information that found although online health information is of variable quality, the majority of it is of reasonably high quality with only a very small proportion considered to be factually incorrect (4/79) or potentially harmful to health (3/79 assessors thought the poor information given may lead someone to “act in a way that may put their health at risk”). Only 3 assessors awarded a discussion thread the lowest rating band overall, whereas 25 assessments rated a message thread in the highest band. This is broadly consistent with previous assessments about the quality of online health information in general [5,10,15,16,45].

### Conclusions

The results of this study suggest that discussion forums are capable of producing health information of reasonably high quality. Of the 79 threads, 68 were assessed to contain at least some medically/scientifically accurate information and 61 of 79 were considered to contain at least some of the medical information that would be expected.

On only 3 occasions did an assessor think someone might make a “somewhat” or “very” ill-advised decision based on the information provided and there were only 3 occasions in which assessors felt the questioner may be led to act in a way that could put their health at risk. In each case, only one of the assessors felt this way when others did not; in the case of 2 of the 3 lowest ratings, comments made in the discussion forum by the original poster could be interpreted as meaning that they were not going to take a potentially harmful course of action. This challenges the assumption that the presence of poor information is automatically harmful.

The forums that contained the most inaccurate or controversial information also contained counterbalancing comments that appear able to dilute the potentially harmful consequences of the poor quality information. Comments made by the original poster and the majority of the respondents suggest that the better quality information was the more influential. This, in particular, warrants further study.

Online discussion forums do seem to be able to provide an opportunity for online health information seekers to access health information of acceptable quality. The findings suggest that there is merit in further exploring the possibilities of online discussion forums for providing peer-to-peer health information. In particular, there is a need to develop a better understanding of whether, and how, the small amount of incorrect or ill-advised information provided in a minority of answers is likely to result in adverse health outcomes or whether the discussion forum characteristics enable such messages to be counteracted and diluted. Most previous studies have tended to hone in on the small minority of poor quality examples and overemphasize the potentially detrimental impact they may have. This is despite the small number of studies that have found evidence of actual harm caused by poor health information found on the Internet. Thus, further analysis of the relationship between poor information and patient interpretation/action is crucial.

## Appendix

Multimedia Appendix 1 Supplementary File 1: Example Assessment Questionnaire.

Multimedia Appendix 2 Data from all survey results including links to the actual question as it appeared on the discussion forum website (as the assessors saw it when they made their assessment).

## Abbreviations

A&E: accident and emergency
AIDS: acquired immune deficiency syndrome
GP: general practitioner
HIV: human immunodeficiency virus
