# Supplementary material for: Health Advice from Internet Discussion Forums: How Bad Is Dangerous?
Source: J Med Internet Res. 2016 Jan 6;18(1):e4. doi: 10.2196/jmir.5051 (PMC4720952; doi:10.2196/jmir.5051)
Supplement: Multimedia Appendix 1 [file jmir_v18i1e4_app1.pdf]

The role of voted discussion forums in providing access to health information

Contact: Jennifer Cole, Jennifer.cole.2013@live.rhul.ac.uk

For website:____

For discussion thread:____

Q1. Did you find the discussion forum answers **[to this question]** to be:

- ○ A. Entirely medically/scientifically accurate
- ○ B. Mostly medically/scientifically accurate
- ○ C. Neither predominantly medically/scientifically accurate nor inaccurate
- ○ D. Somewhat medically/scientifically inaccurate
- ○ E. Very medically/scientifically inaccurate

Please give some comments on your answer:

Q2. Did you find the discussion forum answers **[to this question]** to:

- ○ A. Cover <u>all</u> of the medical/scientific information you would expect to see
- ○ B. Cover <u>most</u> of the medical/scientific information you would expect to see
- ○ C. Cover <u>only some</u> of medical/scientific information you would expect to see
- ○ D. Cover <u>very little</u> of the medical/scientific information you would expect to see
- ○ E. Cover <u>none</u> of the medical/scientific information you would expect to see

Please give some comments on your answer:

Q3. Did you consider the discussion forum answers **[to this question]** to be:

- A. Very sensible
- B. Somewhat sensible
- C. Neither predominantly sensible nor ill-advised
- D. Somewhat ill-advised
- E. Very ill-advised

Please explain your answer:

Q4. Based on the answers given, do you think the poster, or someone reading the discussion thread for advise is most likely to:

- A. Make the most appropriate decision for the medical condition being discussed?
- B. Make a somewhat appropriate decision for the medical condition being discussed?
- C. Be unable to make a decision based on the information provided?
- D. Make a somewhat inappropriate decision for the medical condition being discussed?
- E. Make a very ill-advised decision for the medical condition being discussed?

Please explain your answer:

Q5. How did you find the overall usability of the discussion forum?

- A. It was very easy to follow discussions
- B. It was mostly easy to follow discussions
- C. It was neither particularly easy nor particularly difficult to follow discussions
- D. It was somewhat difficult to follow discussions
- E. It was very difficult to follow discussions

Please explain your answer:

Q6. Based on what you have seen here, do you feel that,

- ○ A. Someone posting on this forum is likely to get a very useful answer?
- ○ B. Someone posting on this forum is likely to get a somewhat useful answer?
- ○ C. Someone posting on this forum is likely to get an answer that will give confusing advice from which it will be difficult for them to make an informed decision.
- ○ D. Someone posting on this forum is likely to get an answer that is unlikely to be particularly helpful?
- ○ E. Someone posting on this forum is likely to get an answer that may lead them into acting in a way that may put their health at risk?

Please explain your answer:

Your name:

Your position relative to the study: GP / Specialist / Patient

If you would be willing to provide us with your contact details, so that we can contact you again if we have any further questions, please enter them below:

Your contact details:

Are you happy for us to contact you again for follow-up information

Would you like us to send you the results of the study?

Have you personally ever used any of the websites we are studying:

a) Y/N (which ones?)

a) If yes, for any topic (not necessarily health-related)

b) for health related topics (if yes, what?)
