# Supplementary material for: Health Advice from Internet Discussion Forums: How Bad Is Dangerous?
Source: J Med Internet Res. 2016 Jan 6;18(1):e4. doi: 10.2196/jmir.5051 (PMC4720952; doi:10.2196/jmir.5051)
Supplement: Multimedia Appendix 2 [file jmir_v18i1e4_app2.pdf]

Multimedia Appendix 2. Data from all survey results including links to the actual question as it appeared on the discussion forum website (as the assessors saw it when they made their assessment).

| | | | Accurate | | | | | Complete | | | | | Sensible | | | | | Appropriate | | | | | Useful | | | | |
|---|---|---|---|---|---|---|---|---|---|---|---|---|---|---|---|---|---|---|---|---|---|---|---|---|---|---|---|
| | | | 1 | 2 | 3 | 4 | 5 | 1 | 2 | 4 | | 5 | 1 | 2 | 3 | 4 | 5 | 1 | 2 | 3 | 4 | 5 | 1 | 2 | 3 | 4 | 5 |
| **DIABETES** | | | | | | | | | | | | | | | | | | | | | | | | | | | |
| Reddit | | | | | | | | | | | | | | | | | | | | | | | | | | | |
| Reddit | Q1. First party since being diagnosed, need advice? (www.webcitation.org/6bvUOZ5dR) | M1 | | | | | | | | | | | | | | | | | | | | | | | | | |
| | | M2 | | | | | | | | | | | | | | | | | | | | | | | | | |
| | | P9 | | | | | | | | | | | | | | | | | | | | | | | | | |
| | Q2. Advice for exercise and midnight lows? | M1 | | | | | | | | | | | | | | | | | | | | | | | | | |
| | (www.webcitation.org/6bvUXCERT) | M2 | | | | | | | | | | | | | | | | | | | | | | | | | |
| Mumsnet | | | | | | | | | | | | | | | | | | | | | | | | | | | |
| | Q3. Are anger outbursts normal with diabetes? | M1 | | | | | | | | | | | | | | | | | | | | | | | | | |
| | (http://www.webcitation.org/6bvUdtbuJ) | M2 | | | | | | | | | | | | | | | | | | | | | | | | | |
| | | P1 | | | | | | | | | | | | | | | | | | | | | | | | | |
| | | P9 | | | | | | | | | | | | | | | | | | | | | | | | | |
| | Q4. Signs of diabetes or paranoid Mummy? | M1 | | | | | | | | | | | | | | | | | | | | | | | | | |
| | (www.webcitation.org/6bvUkaBN5) | M2 | | | | | | | | | | | | | | | | | | | | | | | | | |
| | | P1 | | | | | | | | | | | | | | | | | | | | | | | | | |
| Patient.co.uk | | | | | | | | | | | | | | | | | | | | | | | | | | | |
| | Q5. Longer to get over a cold with diabetes? | M1 | | | | | | | | | | | | | | | | | | | | | | | | | |
| | (www.webcitation.org/6bvUspFMw) | M3 | | | | | | | | | | | | | | | | | | | | | | | | | |
| | | P2 | | | | | | | | | | | | | | | | | | | | | | | | | |
| | | P3 | | | | | | | | | | | | | | | | | | | | | | | | | |
| | Q6. Can this be Diabetes? | M1 | | | | | | | | | | | | | | | | | | | | | | | | | |

| | |
|---|---|
| (www.webcitation.org/6bvV0EGNu) | P2 |
| Q7.<br>Do I have Type 1 Diabetes? | M1 |
| (www.webcitation.org/6bvV8A7uf) | M2 |
| | P1 |
| Q8.<br>Diabetes: Advice please? | M1 |
| (www.webcitation.org/6bvVGmHF9) | P2 |
| **HIV/AIDS** | |
| Reddit | |
| Q9.<br>I found out I had HIV not clear about the stage | M1 |
| (www.webcitation.org/6bvVNKmWP) | M4 |
| Q.10<br>HIV and depression | M1 |
| (www.webcitation.org/6bvVUUaGE) | M4 |
| Q11.<br>Question about HIV and personal fitness | M1 |
| (www.webcitation.org/6bvVcV2cJ) | M4 |
| Q12.<br>FAQ: Worried? Risk, testing and anxiety | M1 |
| (www.webcitation.org/6bvVkHeTv) | M4 |
| Mumsnet | |
| Q13.<br>Babysitter has just announced he's HIV positive | M1 |
| (www.webcitation.org/6bvVsvFwg) | M4 |
| Q.14.<br>Question about HIV (and partner) | M1 |
| (www.webcitation.org/6bvW0Myjx) | M4 |
| Q15.<br>Children and HIV | M1 |
| (www.webcitation.org/6bvW7yBY2) | M4 |
| Patient.co.uk | |
| Q16.<br>HIV question | M1 |
| (www.webcitation.org/6bvWFhPDu) | M4 |

| | | | | | | | | | | | | | | | | | | | | | | | | | | |
|---|---|---|---|---|---|---|---|---|---|---|---|---|---|---|---|---|---|---|---|---|---|---|---|---|---|---|
| Q17.<br>HIV infection: intestinal yeast after 4 months? | M1 | | | | | | | | | | | | | | | | | | | | | | | | | |
| (www.webcitation.org/6bvWM70M0) | M4 | | | | | | | | | | | | | | | | | | | | | | | | | |
| **CHICKENPOX** | | | | | | | | | | | | | | | | | | | | | | | | | | |
| Reddit | | | | | | | | | | | | | | | | | | | | | | | | | | |
| Q18.<br>Is this chickenpox? Help!! | M1 | | | | | | | | | | | | | | | | | | | | | | | | | |
| (www.webcitation.org/6bvWRPVHM) | M4 | | | | | | | | | | | | | | | | | | | | | | | | | |
| | M5 | | | | | | | | | | | | | | | | | | | | | | | | | |
| | M6 | | | | | | | | | | | | | | | | | | | | | | | | | |
| | P4 | | | | | | | | | | | | | | | | | | | | | | | | | |
| | P5 | | | | | | | | | | | | | | | | | | | | | | | | | |
| | P6 | | | | | | | | | | | | | | | | | | | | | | | | | |
| Q.19.<br>Did you give your child the chickenpox vaccine? | M1 | | | | | | | | | | | | | | | | | | | | | | | | | |
| (www.webcitation.org/6bvWXngfA) | M4 | | | | | | | | | | | | | | | | | | | | | | | | | |
| | M5 | | | | | | | | | | | | | | | | | | | | | | | | | |
| | M6 | | | | | | | | | | | | | | | | | | | | | | | | | |
| | P4 | | | | | | | | | | | | | | | | | | | | | | | | | |
| | P5 | | | | | | | | | | | | | | | | | | | | | | | | | |
| | P6 | | | | | | | | | | | | | | | | | | | | | | | | | |
| Q20.<br>Chickenpox: Why more dangerous to adults? | M1 | | | | | | | | | | | | | | | | | | | | | | | | | |
| (www.webcitation.org/6bvWdUui2) | M5 | | | | | | | | | | | | | | | | | | | | | | | | | |
| | M7 | | | | | | | | | | | | | | | | | | | | | | | | | |
| Mumsnet | | | | | | | | | | | | | | | | | | | | | | | | | | |
| Q21.<br>Chickenpox: is 5 months too young to expose? | M1 | | | | | | | | | | | | | | | | | | | | | | | | | |
| (www.webcitation.org/6bvWlC747) | M4 | | | | | | | | | | | | | | | | | | | | | | | | | |
| | M5 | | | | | | | | | | | | | | | | | | | | | | | | | |
| | P4 | | | | | | | | | | | | | | | | | | | | | | | | | |
| | P7 | | | | | | | | | | | | | | | | | | | | | | | | | |
| | P8 | | | | | | | | | | | | | | | | | | | | | | | | | |

| | | | |
|---|---|---|---|
| | | | |
| | Q22.<br>Has your child had the chickenpox vaccine? | M1 | |
| | (www.webcitation.org/6bvWxPmIo) | M4 | |
| | | M5 | |
| | | P6 | |
| | | P7 | |
| | Q23.<br>Is it normal to be so very ill with chickenpox? | M1 | |
| | (www.webcitation.org/6bvX4Fxit) | M4 | |
| | | M8 | |
| | | P4 | |
| Patient.co.uk | | | |
| | Q24.<br>Should toddler get the chickenpox vaccine? | M1 | |
| | (www.webcitation.org/6bvXCED3Q) | M4 | |
| | | M5 | |
| | Q25.<br>Strange symptom with chickenpox | M1 | |
| | (www.webcitation.org/6bvXI8uX2) | M4 | |
| | | M5 | |
